# Assessment in competency-based medical education for anesthesiology: validity evidence, equity, and faculty readiness

**DOI:** 10.3389/fmed.2026.1920129

**Published:** 2026-09-28

**Authors:** Feng Zhou, Qinye Shi, Yeliu Fu, Jiangwei Zhou, Tao Chen, Jianhong Xu

**Affiliations:** 1 Department of Anaesthesiology, The Fourth Affiliated Hospital of Zhejiang University School of Medicine, International School of Medicine, International Institutes of Medicine, Zhejiang University, Yiwu, China; 2 Department of Clinical Nutrition, The Fourth Affiliated Hospital of Zhejiang University School of Medicine, International School of Medicine, International Institutes of Medicine, Zhejiang University, Yiwu, China; 3 Department of Gastroenterology, The Fourth Affiliated Hospital of Zhejiang University School of Medicine, International School of Medicine, International Institutes of Medicine, Zhejiang University, Yiwu, China

**Keywords:** anesthesiology, assessment equity, competency-based medical education, entrustable professional activities, faculty development, milestones, rater training, workplace-based assessment

## Abstract

Competency-based medical education (CBME) is driving a paradigm shift in anesthesiology graduate medical education, moving from fixed-duration training to assessment-centered models. This narrative review aims to synthesize the sources of validity evidence for existing assessment tools (milestones, entrustable professional activities, and workplace-based assessments), equity threats during evaluation and their intervention strategies, and the impact of faculty readiness on assessment quality. Informed by the Scale for the Assessment of Narrative Review Articles as a quality-appraisal instrument, we searched PubMed, Web of Science, CNKI, and Wanfang databases through June 2026. Two authors screened sources and extracted data through iterative team discussions, using a framework approach for thematic organization. Current evidence indicates that workplace-based assessment tools have been validated primarily through single-center, small-sample designs with limited longitudinal outcome data. Generalizability theory analyses demonstrate that acceptable reliability can be achieved with a rational design; however, rater variation remains the predominant source of measurement error. With respect to equity, national studies have identified differential rating growth trajectories among underrepresented-in-medicine residents and female residents, although tool structuration may moderate bias effects. Faculty readiness appears to be a major implementation bottleneck, as most clinical teachers lack formal CBME training. However, anesthesiology-specific evidence is limited and partly relies on cross-specialty surveys. The Chinese elite teaching hospital consortium has proposed a localized milestone evaluation framework, but it remains in early pilot stages. The success of CBME assessment in anesthesiology relies on dynamic balance among tool validity, assessment equity, and faculty readiness, with the interaction of these three domains producing emergent system-level behaviors. Future research should prioritize tracking longitudinal predictive evidence and conducting specialty-specific randomized trials of equity interventions.

## 1 Introduction

Anesthesiology graduate medical education (GME) has historically followed fixed-duration training models (1), where residents progress upon completion of prescribed rotations without systematic verification of clinical competence (2). The limitations of this paradigm are particularly pronounced in anesthesiology (3): some residents complete training without achieving independent practice competency, while high-performing residents are prevented from early graduation by institutional constraints (4, 5). Competency-based medical education (CBME) has emerged as an alternative framework, focusing on the learner and assessing knowledge application in authentic clinical contexts (6, 7). After more than five decades of evolution—from early outcome-oriented concepts to the 2014 Canadian Competence by Design (CBD) reform and the 2020 Accreditation Council for Graduate Medical Education (ACGME) Anesthesiology Milestones 2.0 (8, 9)—CBME has achieved international consensus. Milestones 2.0 subdivides six competencies into “specialty” and “common” categories, simplifies rating scales, and adds behavioral anchors (9).

Within the CBME framework, assessment replaces fixed duration as the central mechanism driving the training progression (10, 11). The ACGME Milestones system employs the Dreyfus five-level framework (Levels 1–5), with Level 4 as the graduation standard (9). Miller's Pyramid of Clinical Competence emphasizes that assessments must be anchored to observable behaviors in authentic clinical settings (12). Workplace-based assessment (WBA) through direct observation of resident clinical performance has become the principal means of CBME implementation in anesthesiology (13). Cross-regional comparisons revealed a substantial overlap among major CBME assessment frameworks (14). Buleon et al. reported that 93% of competency items were shared across the ACGME, CBD, and UEMS/ESAIC standards (14). However, tool convergence does not equate to quality. Rater variation, implicit bias, and structural inequality remain shared challenges across the systems (15, 16).

China's Standardized Residency Training (SRT) system trains more than 100,000 residents annually; however, assessment has long relied on summative written and procedural examinations, with formative evaluation tools remaining scarce (17). In 2018, Peking Union Medical College Hospital, in collaboration with eight teaching hospitals, published the Consensus Framework for Core Competencies for Residents. In 2022, the consortium proposed a milestone evaluation system, formally published in 2023 (17), marking China's entry into anesthesiology residency transformation. Several structural features distinguish the Chinese experience from North American and European frameworks (detailed in Section 3.2). Nevertheless, existing reviews have notable limitations. Weller et al. (5) conducted a systematic review with a literature search ending in 2019, thus excluding post-Milestones 2.0 validation studies and emerging equity discourse. Matava et al. (16) were restricted to a single institution. Faculty readiness has primarily been addressed as background context, with geographic coverage focused on North America and Europe, with limited discussion of CBME initiation strategies in resource-constrained environments.

This review addresses three research questions: (1) What is the level of psychometric evidence and sources of validity evidence for existing CBME assessment tools? (2) What equity threats exist during assessment, and what is the evidence base for intervention strategies? (3) How does faculty readiness influence assessment quality, and what gaps exist in current training systems? These three dimensions form an interconnected assessment quality ecosystem (Figure 1). Assessment tools provide the technical foundation, assessment equity ensures that measurement outcomes are not systematically distorted by demographic characteristics, and faculty readiness determines whether the tools can be implemented correctly. The interaction of these three domains results in emergent system-level behaviors, such as assessment culture, institutional trust, and learner psychological safety, which cannot be fully predicted from any single domain in isolation.

**Figure 1:**
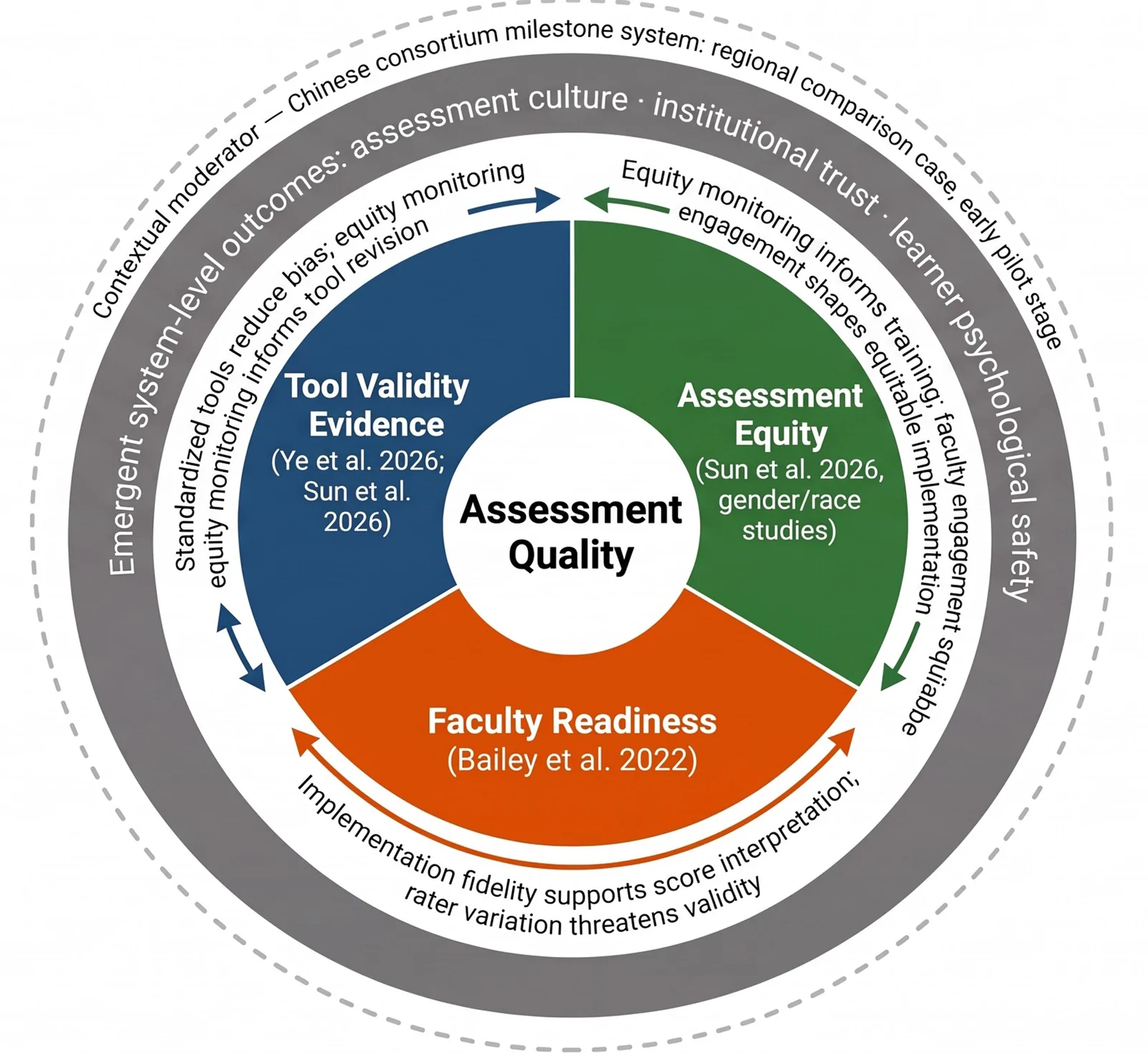
Conceptual model of the Assessment Quality Ecosystem. The three domains interact reciprocally through feedback loops (bidirectional arrows): standardized tools reduce bias while equity monitoring informs tool revision (validity–equity loop); equity monitoring informs faculty training while faculty engagement shapes equitable implementation (equity–readiness loop); implementation fidelity supports score interpretation while rater variation threatens validity (readiness–validity loop). These interactions produce emergent system-level outcomes (outer ring): assessment culture, institutional trust, and learners’ psychological safety. The dashed outer ring denotes the contextual moderator: the Chinese elite teaching hospital consortium milestone system, which was included as a regional comparison case in the early pilot stage. This figure was created with the assistance of the generative AI image tool Jova.ai (https://jova.ai/) and subsequently redrawn, annotated, and finalized using Adobe Illustrator 2024. The authors assume full responsibility for the scientific content and accuracy.

This review focuses on anesthesiology and closely related perioperative specialties—defined as surgical disciplines, critical care, emergency medicine, and pain medicine that share procedural assessment characteristics with anesthesiology—in GME, excluding undergraduate medical education and non-physician training programs (e.g., nurse anesthetists, and anesthesia assistants). Evidence from more distant specialties (e.g., radiology, dermatology, gastroenterology, internal medicine, pediatrics, psychiatry, obstetrics and gynecology, and veterinary education) was included only when methodologically or conceptually informative for anesthesiology assessment design. Such cross-specialty evidence is explicitly identified as an analog at first use, and its applicability to anesthesiology is noted to require prospective validation. The Chinese elite teaching hospital consortium's milestone evaluation system was included as a regional comparison case, with explicit acknowledgment that this system remains in the early pilot stages and its psychometric properties and predictive evidence await multi-center validation.

## 2 Materials and methods

To map the breadth of evidence across three thematic domains, we conducted an initial broad search in PubMed, Web of Science, CNKI, and Wanfang databases through June 2026, using combinations of the following terms: “competency-based medical education,” “CBME,” “anesthesiology,” “assessment,” “workplace-based assessment,” “milestones,” “entrustable professional activities,” “faculty development,” “equity,” and “validity.” This initial search was supplemented by citation snowballing (forward and backward tracking of seminal articles) and expert consultation to ensure that the concept was saturated. Two authors (FZ and QS) engaged in iterative team discussions to evaluate the relevance of the identified sources, with a third author (JX) consulted when the thematic boundaries were unclear. Sources were selected purposively based on their capacity to illuminate tool validity evidence, assessment equity, or faculty readiness in anesthesiology graduate medical education. Cross-specialty evidence was included only when it was methodologically or conceptually informative for anesthesiology assessment design, with its analogical status explicitly noted at first use.

As a narrative review, this work does not claim to exhaustively retrieve systematic data. Instead, it employs purposive sampling to identify conceptually and methodologically informative sources across the three thematic domains. This review synthesizes evidence from heterogeneous sources, including national retrospective cohorts, single-center validation studies, qualitative investigations, and cross-specialty implementation reports. Findings derived from multiple anesthesiology-specific studies with large sample sizes are presented with higher inferential weight. Hypotheses generated from isolated studies, pilot reports, or cross-specialty analogs are explicitly identified as requiring prospective validation in anesthesiology. This review was guided by the Scale for the Assessment of Narrative Review Articles (SANRA) as a quality-appraisal instrument (18), rather than as a comprehensive reporting guideline. A framework approach organized the findings into three themes: tool validity evidence, assessment equity, and faculty readiness.

## 3 Historical evolution and theoretical frameworks

### 3.1 From time-based to competency-based paradigm transition

Anesthesiology residency training has long used a fixed duration as the progression standard (1, 4, 5, 19). CBME transforms assessment from a summative judgment at the completion of training to a formative mechanism that drives the entire training process (20).

The United States and Canadian systems constitute the dominant CBME assessment paradigms. The ACGME launched Milestones 1.0 in 2013 and Milestones 2.0 in 2020 (9, 20, 21); the Royal College of Physicians and Surgeons of Canada initiated the Competence by Design reform in 2014 (22). European, British, and Australasian systems offer differentiated pathways (23–25), with the Australian and New Zealand College of Anesthetists implementing competency-based training in 2012 and demonstrating through 67,405 WBA records that 12 assessments achieve a decision reliability of 0.70 (25). Cross-regional comparisons demonstrate convergence in core tools, yet significant differences in training duration, technological platforms, and unique designs (Table 1). These gaps constitute the starting point for this review.

**Table 1:** Cross-regional comparison of major anesthesiology CBME assessment frameworks.

Dimension	ACGME (USA)	CBD (Canada)	EBA/UEMS (Europe)	RCoA (UK)	ANZCA (Aus/NZ)
Initiation year	2013 (Milestones 1.0)	2014 (CBD)	2020 (CBMET Consensus)	2021 (Curriculum Update)	2012
Core tools	Milestones + EPAs + WBA	EPAs + WBA	Milestones + WBA (recommended)	SLEs (A-CEX, CbD, MSF, MTRs) + WBA	Milestones + EPAs + WBA (mini-CEX, DOPS, CbD, MSF)
Rating framework	Dreyfus 1–5	Dreyfus 1–5	Dreyfus 1–5 (recommended)	Dreyfus 1–5	Dreyfus 1–5
Training duration	4 years (CA-1 to CA-3 + research year)	5 years	5 years (including 1 year ICU)	7–8 years (including core training)	5 years
Technical platform	ADL Milestones/institution-specific	One45/MedSIS/institution-specific	No unified platform	LLP (Lifelong Learning Platform)	TPS (Training Portfolio System)
Formative assessment proportion	High (∼60%–70%)	High (∼60%–70%)	Variable (cross-country differences)	High (∼50%–60%)	High (∼70%–80%)
Unique design features	Milestones 2.0 simplified levels + behavioral anchoring	Hybrid time-competency model	Cross-national consensus; large implementation variation	Diversity/inclusivity cultural values	TPS real-time tracking; entrustment-supervision scale; rigorous MSF design (≥15 colleagues, ≥ 8 valid feedback responses)
Validation evidence	Extensive (multi-center, >10 years)	Extensive (nationwide implementation)	Limited (cross-country variation)	Moderate (2021 new curriculum)	Extensive (67,405 WBA records)

Proportions of formative assessment and characterizations of validation evidence are approximate estimates derived from the published curriculum documents, consensus statements, and validation studies cited in Sections 3 and 4, intended for comparative orientation rather than precise quantification.

### 3.2 Early explorations by the Chinese elite teaching hospital consortium

China's Standardized Residency Training system trains more than 100,000 residents annually; however, assessments have long relied on summative written and procedural examinations with insufficient formative evaluation tools. In 2018, Peking Union Medical College Hospital, together with eight teaching hospitals, published a Consensus Framework for Resident Core Competencies. In 2022, the consortium proposed a milestone evaluation system, formally published in 2023 (17), marking China's entry into the transformation of anesthesiology residency. This system divides the six competencies into common and specialty components, with “professionalism” encompassing integrity, empathy, and legal compliance—dimensions that incorporate local sociocultural elements beyond the ACGME framework.

Several structural features distinguish the Chinese experience from North American and European frameworks. First, administrative integration within hospital hierarchies enables rapid pilot deployment but limits cross-institutional standardization. Second, professionalism indicators explicitly incorporate integrity and legal compliance dimensions that reflect local sociocultural values. Third, the scale of more than 100,000 annual trainees across heterogeneous sites constrains quality assurance mechanisms. Cui et al. (26) employed behavioral event interviewing and the Delphi method, involving 15 experts and conducting two rounds of consultation (Kendall's concordance coefficient W = 0.312, *P* < 0.05) to construct an evaluation framework that includes six primary indicators and 23 secondary indicators. This study was limited to a single region, restricting external validity. The consortium system is currently piloted in eight hospitals and has not yet developed into a national standardized instrument.

Beyond the consortium system, other Chinese teaching hospitals have conducted independent CBME assessments. West China Hospital of Sichuan University constructed a “six-in-one” residency system, establishing hospital-specific five-stage evaluation standards for six core competencies, integrating process assessment, 360-degree evaluation, and credit-based clinical course grades. Peking University Third Hospital developed a competency-oriented hierarchical progressive training system, resulting in a 15.00 ± 5.89 point (*P* < 0.05) increase in resident self-assessed competency scores, and annual examination pass rates improved from 83% to 100% (27). The Central Theater Command General Hospital of the Chinese People's Liberation Army developed a competency evaluation index system comprising six primary and 24 secondary indicators, with an overall Cronbach's *α* of  0.840 and a KMO of  0.759. Since 2013, Xiangya Hospital of Central South University has adopted Yale University's medical education training philosophy, establishing six training objectives, including GAF and Mini-CEX formative assessment tools, and implementing a “stepwise” training model.

These explorations collectively indicate that Chinese anesthesiology residency is transitioning from summative examination to formative evaluations, yet each system remains independently developed without unified standards or cross-institutional validation. Unlike the centralized national frameworks in North America and Europe, the Chinese model is characterized by hospital-level autonomy, rapid localized adaptation, and limited psychometric validation. This model may serve as a reference for healthcare systems with limited resources and significant administrative inertia, but its effectiveness awaits longitudinal tracking and outcome validation.

## 4 Assessment tools: validity evidence and psychometric foundations

To systematically present the theoretical foundations, sources of validity evidence, and evidence gaps of CBME assessment tools in anesthesiology, this review developed an integrated framework diagram (Figure 2). The specific sources of validity evidence for each tool are detailed below.

**Figure 2:**
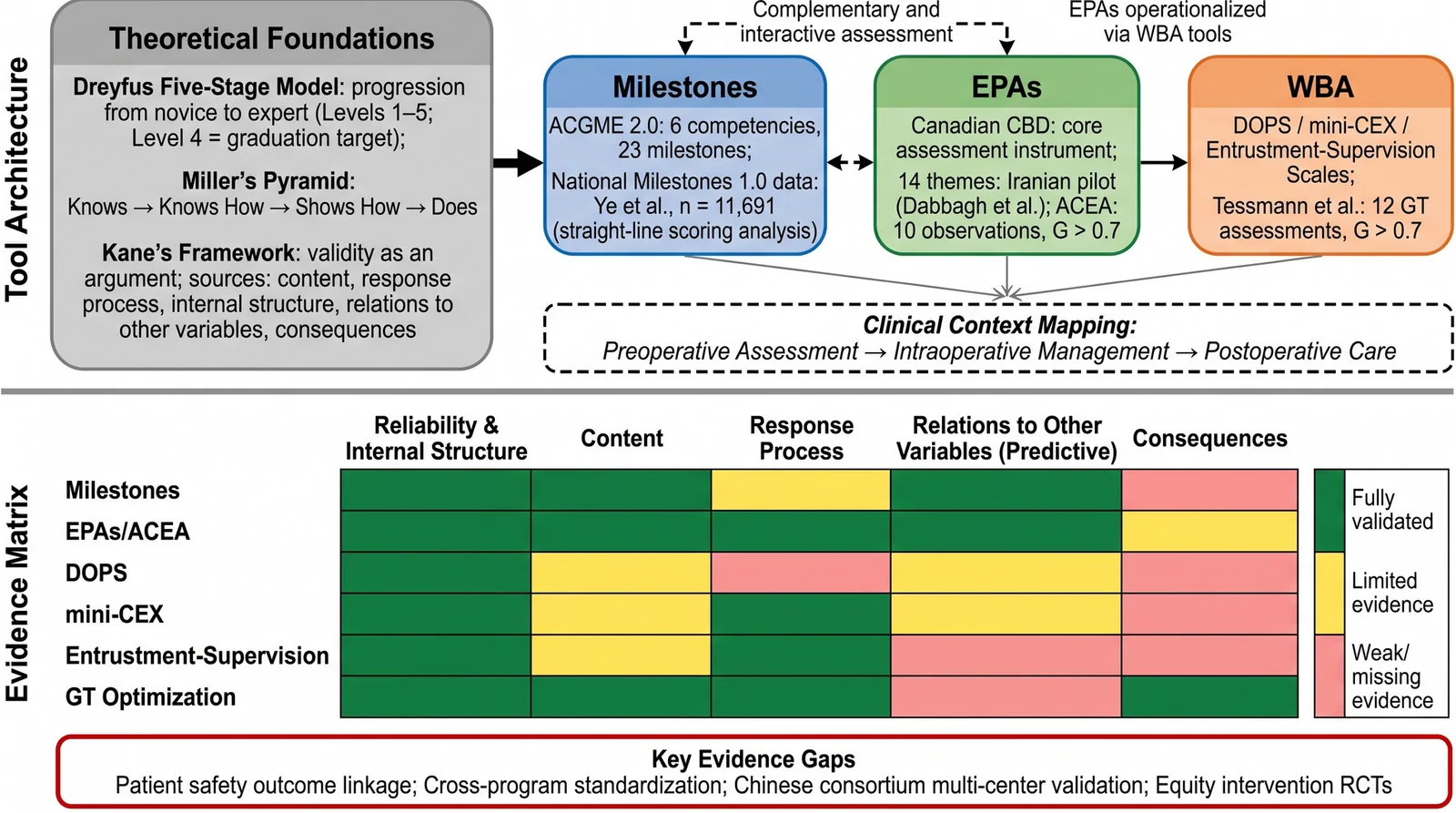
Integrated framework of validity evidence and psychometric foundations for CBME assessment tools in anesthesiology. Upper panel: theoretical foundations (Dreyfus five-stage model, Miller's pyramid, and Kane's argument-based framework) supporting the three core assessment tool families—Milestones, entrustable professional activities (EPAs), and workplace-based assessment (WBA)—and their mapping to clinical contexts (preoperative assessment, intraoperative management, and postoperative care). Lower panel: evidence matrix summarizing the level of validity evidence for each tool across five sources of validity evidence [reliability and internal structure, content, response process, relations to other variables (predictive), and consequences]; green indicates strong evidence, yellow indicates limited evidence, and red indicates weak or absent evidence. The bottom bar lists key evidence gaps requiring priority attention. This figure was created with the assistance of the generative AI image tool Jova.ai (https://jova.ai/) and subsequently redrawn, annotated, and finalized using Adobe Illustrator 2024. The authors assume full responsibility for the scientific content and accuracy.

### 4.1 Milestones and EPAs: complementary frameworks

Milestones and EPAs constitute the two assessment pillars of CBME: milestones track the longitudinal development of competency sub-components (9), while EPAs measure performance on integrated clinical tasks (7). A correspondence between the two has been established: the ACGME Milestones system encompasses pre-anesthetic assessment, intraoperative management, and postoperative care. Canada's CBD uses EPAs as the core assessment instrument (9, 28). An Iranian study developed 14 EPA themes through a 10-step model, achieving correspondence with milestone competency sub-components (29). The Anesthesia Clinical Encounter Assessment (ACEA) tool, developed by the University of Toronto, has been validated through global rating scales (GRS). With 10 independent observations, it achieved the 0.70 reliability threshold (19), providing a reference standard for the reliability of the WBA tool.

Recent national cohort studies have raised important questions about the validity of milestone-based assessment in anesthesiology. Ye et al. (21), analyzing Milestones 1.0 data from 11,691 residents across 153 programs, found that 88.9% of programs exhibited at least one instance of straight-line scoring (identical ratings across all 25 sub-competencies in a reporting period), and that residency programs accounted for 63%–84% of between-resident variability in baseline ratings and 60%–92% in growth rates. This suggests that Milestones ratings may reflect program-level evaluation culture more than individual resident progress. Furthermore, Sun et al. (30) compared final Milestones 2.0 ratings with American Board of Anesthesiology certifying examination performance in 1,849 residents. They found only weak correlations (*r* = 0.112–0.135) between average milestone ratings and board examination Z-scores, with no significant associations between conceptually related milestone domains and oral examination task ratings. These findings challenge the assumption that current milestone scoring captures competency domains relevant to independent practice.

### 4.2 Systematic evidence for workplace-based assessment

WBA measures resident performance through direct observation in authentic clinical contexts. Weller et al. (5), in a systematic review covering the literature from nine countries, noted that studies of WBA tool validity are predominantly single-center, small-sample designs lacking longitudinal tracking and patient safety outcome indicators, with the evidence base remaining relatively weak.

WBA tools include Direct Observation of Procedural Skills (DOPS) for technical procedures, the Mini-Clinical Evaluation Exercise (mini-CEX) for comprehensive clinical assessment, and entrustment-supervision scales for supervisory trust judgments (5). Tessmann et al. (31) collected 3,936 EPA assessment records across 28 Swiss training programs. The generalizability theory (GT) analysis showed that achieving a G coefficient >0.7 requires at least three EPA types, each assessed four times by four supervisors, totaling 12 assessments. GT quantifies error sources from raters, occasions, and tasks to optimize design: increasing raters reduces subjective error but increases administrative burden. Increasing occasions capture competency comprehensiveness but extend the cycle. Quantitative analysis indicates that completing one EPA assessment form requires only three minutes on average, with the burden remaining manageable under rational design (32). Although this estimate originates from a radiology residency program, its quantitative methodology is directly applicable to the design of anesthesiology EPA workflows. For programs with insufficient raters, increasing the number of assessment occasions can compensate for rater quantity deficits.

### 4.3 Validity evidence for direct observation of procedural skills

DOPS represents the most technically mature WBA tool. An Iranian study employed repeated DOPS (R-DOPS) to conduct 6-month longitudinal tracking of 18 anesthesiology residents (33), demonstrating improvement in procedural skills, with assessment and feedback requiring approximately 24 min in total (33). However, this study used a single-center design (*n* = 18), lacked longitudinal outcome data (e.g., complication rates), and repeated assessments by the same supervisor may have introduced familiarity effects.

Accumulating validity evidence for DOPS continues to face practical challenges: emergency and elective intubation differ significantly in cognitive load, yet existing tools fail to adequately distinguish these contexts. Feedback quality varies with assessor teaching competence, and tracking periods are typically short (6–12 months), which is insufficient to capture complete learning curves for skills requiring extended practice (e.g., peripheral nerve blocks).

### 4.4 Reliability and validity evidence

With respect to reliability, DOPS demonstrates good internal consistency (Cronbach's *α*) (33), but inter-rater reliability remains problematic (34). Significant variation in rater stringency during anesthesiology EPA assessments may introduce outcome bias. Anchor cases and group calibration meetings can reduce cognitive divergence, but assessor concerns about consequences also diminish reliability (35). The dynamic nature of clinical competence renders single-test-retest inappropriate. Multiple measurements should be conducted over weeks or during a rotation period to analyze change trajectories (31).

For sources of validity evidence, we adopted Kane's argument-based validation framework, which views validity as an evolving argument concerning the interpretation and use of assessment scores within defined contexts (36). Sources of validity evidence include content (expert review and Delphi methods) (29), response process (teacher cognitive load and rating authenticity) (37), internal structure (factor structure and reliability), relations to other variables (convergent and predictive evidence), and consequences (impact on learning behaviors and wellbeing) (38, 39). Content evidence is established through expert review and Delphi methods (29). Response process evidence warrants attention: teachers experience dual pressure from form formats and assessment consequences when completing EPA forms, potentially threatening rating authenticity (37). Consequential evidence requires that the assessment promote learning behaviors. Canadian residents in CBME environments commonly report stress and frustration (38), indicating that assessment design must identify weak areas and provide targeted feedback to guide genuine self-improvement (39). Predictive evidence—defined here as the relationship between assessment scores and subsequent clinical performance outcomes—remains limited for anesthesiology WBA tools, with most studies lacking longitudinal tracking of independent practice competency or patient safety indicators.

## 5 Assessment equity: evidence and interventions

Assessment equity threats originate not from a single source but from the interaction of group-level structural inequality, individual-level rater heterogeneity, and cognitive bias (Figure 3).

**Figure 3:**
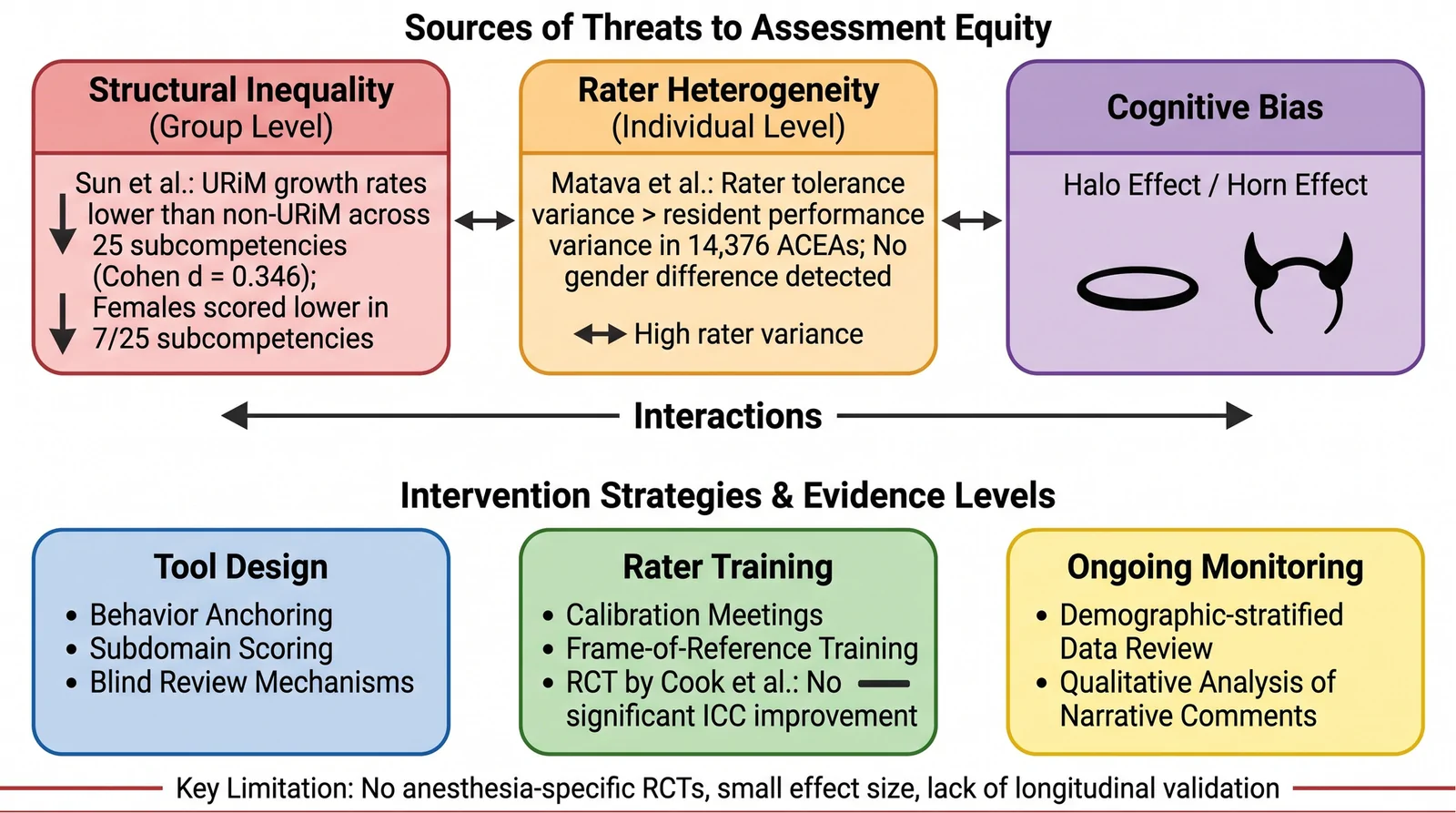
Integrated framework of assessment, equity threat sources, and intervention strategy evidence levels. Upper panel: three interacting sources of threats to assessment equity—group-level structural inequality (slower rating growth trajectories among underrepresented-in-medicine residents and lower ratings for female residents in selected sub-competencies), individual-level rater heterogeneity (rater tolerance variance exceeding resident performance variance, with no detectable gender differences in highly structured tools), and cognitive bias (halo and horns effects). Lower panel: intervention strategies at three levels—tool design (behavioral anchoring, subdomain scoring, and blinded review), rater training (calibration meetings and frame-of-reference training, noting that the randomized trial by Cook et al. found no significant inter-rater reliability improvement), and ongoing monitoring (demographic-stratified data review and qualitative analysis of narrative comments). The bottom bar indicates the key limitation of the current evidence base: no anesthesiology-specific intervention RCTs, small effect sizes, and lack of longitudinal validation. This figure was created with the assistance of the generative AI image tool Jova.ai (https://jova.ai/) and subsequently redrawn, annotated, and finalized using Adobe Illustrator 2024. The authors assume full responsibility for the scientific content and accuracy.

### 5.1 Types and sources of assessment bias

Bias in CBME assessment may distort the judgment of resident competence. Gender bias has received the most research attention. Sun et al. (15) found that female residents received lower ratings on 7/25 sub-competencies. However, Matava et al. (16), in their review of 14,376 ACEA assessments, found no gender differences, suggesting that tool design may moderate bias effects. Racial and non-native English speaker bias also exists, with assessors potentially underestimating clinical communication competence in relevant groups due to cultural differences or language barriers (40, 41). Cognitive bias constitutes another important source: the “halo effect” leads assessors to overestimate other dimensions based on outstanding performance in one dimension. The “horns effect” lowers overall ratings because of negative impressions. Structured tools, such as behaviorally anchored rating scales, reduce the arbitrariness of subjective judgment by translating abstract competencies into specific observable behaviors (42).

### 5.2 Gender, race, and assessment equity: multi-center evidence

Sun et al. (15), in a retrospective cohort study of 5,049 anesthesiology residents, found that underrepresented-in-medicine (URiM) residents exhibited slower rating growth trajectories across all 25 sub-competencies compared to non-URiM residents (Cohen d = 0.346), and female residents received lower ratings on 7/25 sub-competencies (Cohen d = 0.222–0.346). Similar differences have been reported in cross-specialty cohorts. Boatright et al. (43) in internal medicine, Walters et al. (44) in pediatrics, and Landau et al. (45) in general surgery have all noted associations between trainee sex and ACGME milestone assessments. This cross-specialty evidence collectively suggests that assessment equity is not a methodological defect unique to anesthesiology but a shared challenge across CBME assessment systems.

However, Matava et al. (16), in their review of 14,376 ACEA and 4,467 EPA assessments, found no significant differences in entrustment ratings between male and female residents. This “null finding” reveals the moderating effect of assessment system design: the ACEA tool employs GRS with behaviorally anchored descriptions, potentially exhibiting higher structuration than Milestones 1.0's ten-point rating scale, thereby compressing the space for subjective judgment variation. Reasons for Matava et al.'s failure to detect gender differences include sample selection bias (single academic center), tool differences (GRS continuous ratings may dilute gender stereotype effects), and differences in assessment context. ACEA focuses on clinical encounters, whereas Milestones covers broader non-technical competencies. Furthermore, Matava et al. (16) found that rater leniency heterogeneity exceeded resident performance variation, indicating that the primary source of assessment difference lies in individual rater tendencies rather than the true competence of residents. Sun et al. (15) revealed group-level structural inequality, while Matava et al. (16) revealed individual-level rater heterogeneity. Together, these findings constitute dual threats to assessment equity (15, 16).

### 5.3 Methods to reduce assessment bias

Reducing assessment bias requires intervention at three levels: tool design, assessment process, and assessor composition. At the tool design level, behavior-specific anchors should replace abstract descriptions, with rating dimensions refined and itemized scoring required (46). At the assessment process level, “blinded” mechanisms can reduce identity bias. Standardized scenarios and unified benchmarks can minimize environmental variable effects (47). Mobile applications enable real-time, high-frequency data collection, reducing dependence on single assessments (31). At the assessor composition level, diversified assessment panels can evaluate performance from multiple perspectives (48). Calibration meetings, where assessors jointly discuss and rate standardized cases, facilitate unified scoring standards (49).

Nevertheless, empirical evidence for the effectiveness of intervention remains limited. Although conducted in internal medicine, a randomized controlled trial by Cook et al. (50) with 52 outpatient supervisors found no significant difference in inter-rater reliability between frame-of-reference training and delayed training (ICC 0.40 vs. 0.43, *p* = 0.19). Its null finding informs the design of anesthesiology rater training but requires specialty-specific replication. A multi-center cluster randomized controlled study in internal medicine by Carnes et al. (51), involving 8,657 faculty, provides a methodological template for bias-reduction workshops. Their 3-h bias-reduction workshop significantly improved the intervention group’s performance in bias vulnerability awareness (*b* = 0.190, *p* = 0.02), bias-reduction self-efficacy (*b* = 0.097, *p* = 0.03), and taking action to reduce bias (*b* = 0.113, *p* = 0.04). However, effect sizes were small (0.10–0.11), and longitudinal data validating behavioral change persistence were lacking. No study on the effectiveness of interventions specifically targeting anesthesiology CBME assessment currently exists. Sun et al. (15) proposed a three-dimensional intervention framework: rater training, inclusive assessment design, and continuous monitoring. Milestones 2.0, by reducing sub-competency numbers and simplifying language, is regarded as a promising strategy for reducing unnecessary variation.

### 5.4 Cross-Validation and multiple evidence triangulation

Single assessment results cannot comprehensively reflect resident competence and are susceptible to bias threats. Cross-validation enhances conclusion validity and equity through the integration of multiple evidence sources. Self-assessment reveals self-perception. Peer assessment provides teamwork perspectives. Attending assessment focuses on clinical decision-making. Patient feedback focuses on humanistic care (52). Learning portfolios systematically collect procedure logs, reflective journals, and video records to construct longitudinal, dynamic competency development trajectories (46). Introducing standardized patients or simulated scenarios provides uniform assessment benchmarks, controlling environmental variables, and enhancing horizontal comparability (53). Cross-validating simulation assessment results with clinical WBA results enables a more accurate judgment of competency transfer (54).

## 6 Faculty readiness: gaps, training, and systematic development

### 6.1 Faculty readiness gaps

Faculty readiness directly determines assessment quality. Bailey et al. (55), in a national survey of Canadian pediatric anesthesia faculty, found that 83% were familiar with basic CBME concepts; however, 64% had never received formal training, and 52% had never used CBME assessment tools. With regard to training preferences, 72% favored small-group discussions, 62% preferred interactive lectures, and 50% selected workshops. These preferences indicate that faculty development programs must shift from “concept dissemination” to “skill practicum.”

An educational intervention study with 104 psychiatry faculty (56) showed that CBME knowledge training significantly enhanced learner readiness, but faculty readiness improvement did not reach statistical significance. This cross-specialty evidence suggests a universal “knowledge–behavior gap” that likely extends to anesthesiology faculty development, reflecting a bottleneck of effective knowledge transfer and difficult behavior change. Faculty development should not be limited to one-time workshops but must construct continuous longitudinal support systems, including rater calibration workshops, peer mentoring, teaching observation, and feedback loops.

The Dalhousie University Department of Anesthesiology qualitative research identified three groups: innovators/early adopters, early/late majority, and laggards (57). The early/late majority showed the greatest progress during the 1-year tracking, shifting from skeptical optimism to active engagement, suggesting that interventions should target this group. Faculty members have universally expressed concerns about the lack of empirical evidence, demonstrating the superiority of CBMEs over time-based training (57). Raj and Thorn (58) demonstrated that pre-assessment training, which enables teachers to thoroughly understand the Milestones system, through detailed discussion and consensus on competency-level performance, significantly reduced rater error. Clinical Competency Committees (CCC) should comprehensively collect daily work performance and examination grades, using group discussions to synthesize ratings and improve accuracy (59).

### 6.2 Core components of rater training

Rater training must systematically cover three dimensions: assessment theory, tool use, and feedback skills. At the theoretical level, concepts of reliability, validity, and bias are explained to help teachers understand the scientific foundation of assessment (60). When assessment processes are disconnected from clinical contexts, teachers and residents may perceive assessment as “bureaucratic box-ticking” behavior (60).

At the tool use level, focus is placed on standardized interpretation of rating scales and behavioral observation techniques. An emergency medicine faculty training program demonstrated that experiential learning enabled teachers to select context-appropriate EPAs more accurately and effectively record assessment results (61). This procedural specialty provides a conceptual analog for anesthesiology EPA selection training, pending direct validation. Frame-of-reference training, through joint discussion of standardized cases to anchor rating standards, enables teachers from different backgrounds to reach consensus on the same performance. At least 8–12 h of intensive training can effectively improve rating accuracy (61).

At the feedback skills level, teachers must learn to provide specific, constructive diagnostic feedback rather than vague grades. A radiology faculty survey showed that most teachers recognized the importance of high-quality written feedback and were willing to enhance their competence through workshops (62). The reported training preferences align with those of the anesthesiology faculty but require specialty-specific confirmation. Feedback effectiveness depends on the “feedback culture” shaping: when assessment is perceived as bureaucratized, teachers and residents may fall into “box-ticking” behavior, with feedback becoming superficial (60). The residual effects of negative feedback must be balanced through supportive mentoring and individualized learning plans (63).

### 6.3 Systematic development of faculty development systems

Systematic faculty development is the cornerstone of ensuring quality and sustainability assessments. The Clinician-Educator Track, through assessment competency certification and lifelong learning pathways, helps teachers systematically master educational theory, assessment tool development, and feedback techniques (64). The Assessment Quality Committee regularly reviews data distribution characteristics, bias indicators, and rating consistency, providing targeted feedback and retraining for the faculty (60). When teachers are aware that assessment behaviors are subject to systematic monitoring, they are more inclined to adjust their strategies and reduce subjective bias (62).

The Swiss Institute for Medical Education (SIME), following Kern's six-step curriculum development cycle, established a modular training system (65): over 30 workshops were conducted within 2 years, reaching more than 500 clinical teachers, with median satisfaction ratings of 9 (out of 10). The key success factors included rigorous design processes, active expert teams, and tailored content.

In China, SIME experience may serve as a reference. The Chinese Medical Doctor Association's “Competency-Oriented Anesthesiology Residency Faculty Training Program” can function as a seed project that expands into a hierarchical progressive system. The foundation level disseminates CBME concepts and tool use. The advanced level focuses on rater calibration and feedback skills. The leadership level cultivates assessment system design and quality monitoring capabilities. Faculty development systems must also attend to peripheral groups such as community preceptors, providing accessible training through flexible formats, including online modules and workshops (66).

### 6.4 Assessment administrative burden and learner wellbeing

Restructuring CBME assessment, while enhancing effectiveness, significantly impacts resident learning experience and mental health. The 2020 Quebec Resident Federation report (67) indicated that 67% of residents felt frustrated at least weekly due to the administrative burden of CBD, 42% experienced burnout, and 37% reported tremendous pressure from continuous monitoring.

Qualitative research conducted at Dalhousie University with anesthesiology, internal medicine, and surgery residents (68) further revealed that residents described CBD as a “crushing burden.” They reported requiring approximately 2 h weekly to track EPA completion and noted that repeated requests for supervisor assessment potentially damaged working relationships. Day et al. (38), in a qualitative study of 16 Canadian residents, showed that administrative burden led to frustration, anxiety, feeling overwhelmed, and burnout. Braund et al. (69), in a national mixed-methods survey of 375 Canadian residents, provided the largest quantitative evidence to date. CBME groups reported stronger negative emotions, including stress, anxiety, and frustration. Some residents “gamed” clinical experiences to complete EPAs rather than naturally immersing in learning.

These cross-system data collectively suggest that the CBME administrative burden's impact on learner mental health is universal. Assessment system design must incorporate learner wellbeing as a core consideration, avoiding formalism that “assesses for assessment's sake” and erodes clinical learning enthusiasm.

A nascent but relevant construct in this context is “assessment fatigue,” which is conceptually distinct from general burnout and specifically attributable to the cumulative cognitive load of continuous performance evaluation. As EPA and WBA volumes expand in anesthesiology residency, this construct may become increasingly relevant and warrant monitoring in future implementation research.

## 7 Current challenges and research gaps

### 7.1 Insufficient sensitivity of assessment tools for high-stakes procedures

Existing assessment tools exhibit limited sensitivity in capturing competency differences during crisis events, such as difficult airway management and malignant hyperthermia. Traditional checklist-based scoring tends to produce ceiling or floor effects, making it difficult to differentiate between senior residents and experts, or to detect subtle progress among novices (70). While the ACEA tool demonstrates good reliability and validity for perioperative routine competency assessment, its global rating scale (GRS) requires at least 10 independent observations to achieve the 0.70 reliability threshold when distinguishing subtle performance differences across training stages (19). Cumulative sum (CUSUM) analysis has been applied to competency assessment for ultrasound-guided venous access procedures to identify learners failing to meet expected progress (71). However, it has not been widely integrated into daily assessment systems, and its cross-scenario generalizability and predictive evidence are still awaiting verification.

### 7.2 Standardization and generalizability of multi-site assessment

Anesthesiology training programs are distributed across sites with varying scales and resource conditions, and case mix, faculty level, and patient population differences cause systematic bias in the same tool. A 39-country European survey showed training duration ranging from 24 to 84 months, directly affecting cross-site comparability (72). In China's Standardized Residency Training, only 3.9% fully understand CBME training objectives and assessment systems (73), highlighting inconsistent information transmission and standard execution during multi-site implementation.

Shared assessment databases and technology platforms are strategies for enhancing comparability. The University of Toronto ACEA tool collected 8,664 records from 342 assessors through an online platform, and a generalizability analysis determined the minimum observation count required for reliable ratings (19). Mobile applications (e.g., prEPAred app) record EPA supervision levels in real time, promoting cross-site immediacy and consistency (31). However, technology dissemination faces resource allocation inequality barriers, with faculty shortages and limited infrastructure in low- and middle-income countries constraining its popularization (74).

### 7.3 Emerging technology-assisted assessment: opportunities and caution

Empirical evidence supports limited applications of AI in assessment workflow organization. Booth et al. (75), in a multi-program study, used natural language processing to automatically classify 5,935 narrative assessments into ACGME Milestones 2.0 sub-competencies, achieving an AUC of 0.79 for professionalism and interpersonal communication domains. This demonstrates AI's potential efficiency in organizing large feedback volumes, although the study was restricted to four programs and did not evaluate the impact on rater decision-making or learner outcomes. Technology integration more broadly faces privacy protection, equipment cost, learner acceptance, and algorithmic bias barriers (76).

Emerging technological possibilities reported during 2025–2026 include generative AI applications in automated scoring (77) and personalized feedback generation (78). These applications remain experimental. Seneviratne et al. (77) reported a high correlation between AI and human examiner markings for short-answer questions, but the study was limited to a single institution and a narrow question format. Schaye et al. (78) developed an LLM-based clinical reasoning assessment tool across two institutions; however, its generalizability to anesthesiology procedural competency assessment has not been tested.

Beyond these isolated studies, the uncritical AI adoption introduces new validity threats. The “low-skill overestimation” bias, wherein AI-generated scores inflate estimates for low-performing learners, may systematically mask true competency gaps. AI should therefore be positioned as a “supportive tool” rather than a “replacement solution.” We propose the Generative-Evaluative Agreement (GEA) framework as a conceptual validity checkpoint for AI-assisted assessment. GEA is defined as the systematic comparison of AI-generated scores or feedback with human evaluator judgments, using predefined agreement thresholds and discrepancy resolution protocols. This framework is derived from existing validity theory and human-machine collaboration literature, but is an author-generated conceptual model that has not been independently validated. Its operationalization and effectiveness in anesthesiology CBME require prospective empirical testing before incorporation into high-stakes decisions.

### 7.4 Integrative synthesis: ecosystem dynamics

The three domains reviewed above—tool validity evidence, assessment equity, and faculty readiness—do not function in isolation. Rather, they interact reciprocally to produce emergent system-level behaviors that are not fully predictable from any single domain in isolation (Figure 1). Inadequate faculty readiness increases rater variation, which simultaneously threatens tool validity (by inflating measurement error) and assessment equity (by introducing systematic bias against underrepresented groups). Conversely, when equity concerns are unaddressed, faculty engagement may decline, further compromising implementation quality and perpetuating a cycle of distrust. These feedback loops generate emergent properties, including assessment culture, institutional trust, and learner psychological safety, that constitute the true test of CBME sustainability.

The reviewed evidence illustrates this dynamic. Ye et al. (21) and Sun et al. (30) demonstrated that milestone ratings reflect the program-level evaluation culture more than individual competence, suggesting that faculty readiness deficits have corrupted the validity argument for milestone-based decisions. Sun et al. (15) and Matava et al. (16) collectively revealed that assessment differences originate from both group-level structural inequality and individual-level rater heterogeneity, indicating that equity and validity threats are co-dependent rather than independent. Bailey et al. (55) showed that 64% of pediatric anesthesia faculty had never received formal CBME training, with the resulting rater variation constituting a shared root cause for both validity attenuation and equity distortion. The Chinese consortium system (17, 26, 27) further exemplifies how rapid tool deployment without parallel faculty development and equity monitoring produces pilot systems with uncertain psychometric properties. Future implementation strategies must therefore address the three domains simultaneously, rather than sequentially, to achieve dynamic equilibrium.

## 8 Future directions and recommendations

### 8.1 Rater training systems

Faculty readiness appears to be a significant implementation bottleneck for assessment quality, although current anesthesiology-specific evidence remains limited and draws partly on cross-specialty surveys (55). Future efforts should institutionalize multi-level faculty development programs that integrate three modules: rater calibration workshops (standardized video cases), feedback skills training (specific, behavior-oriented), and implicit bias recognition modules. Program design should prioritize interactive formats and establish rater performance monitoring mechanisms (57, 58).

### 8.2 Assessment architecture simplification

The CBME administrative burden has become a significant threat to learner wellbeing. Recommendations include reducing EPA quantity, simplifying documentation requirements, and improving technology interfaces, while maintaining validity and reducing burden. Competency committees should enhance transparency, avoiding “black-box decision-making” that erodes learner trust and autonomy (68, 79). Entrustment-supervision scales should be prioritized, given their higher clinical relevance and greater convenience.

### 8.3 Equity safeguards

Sun et al. (15) proposed a three-dimensional intervention framework comprising rater training, inclusive assessment design, and continuous monitoring.

At the rater training level, ACGME provides structured training for CCC members to establish shared mental models of Milestones and consistent rating standards, with faculty training further embedding implicit bias recognition modules (57, 80). At the assessment tool level, programmatic assessment integrating multiple low-stakes formative evaluations is superior to single high-stakes assessments, better capturing true competence and reducing bias opportunities (15). At the institutional monitoring level, regular demographic decomposition of assessment data is recommended, with a shift toward narrative comment qualitative analysis to understand how bias manifests in rater language expression.

### 8.4 AI-assisted assessment

Based on the emerging technological possibilities and validity concerns outlined above, we offer three author-generated recommendations for the cautious integration of AI. First, pilot AI-generated personalized feedback should be implemented exclusively for formative assessments, while maintaining supervisor authority for final review. Second, large language model bias recognition training should be introduced in rater calibration workshops to enable supervisors to understand algorithmic bias sources. Third, “human–machine collaboration” workflows should be established, with AI responsible for data integration and trend analysis, and human raters responsible for contextual judgment and ethical decision-making.

The proposed GEA framework should serve as a conceptual validity checkpoint—requiring systematic comparison between AI-generated and human evaluator judgments with predefined agreement thresholds—before AI-assisted scoring is considered for high-stakes decisions. The operationalization and effectiveness of GEA in anesthesiology CBME requires prospective empirical validation.

### 8.5 Chinese consortium system validation

The milestone evaluation system of the Chinese elite teaching hospital consortium is in early pilot stages. Future directions include the following: (1) developing anesthesiology-specific milestone evaluation indicators with clear competency level descriptions for each training stage; (2) establishing supporting evaluation tool portfolios; (3) standardizing CCC operational procedures; and (4) conducting multi-center validation studies to verify predictive clinical outcome validity and assessment equity.

### 8.6 Research evidence-based

Current CBME assessment research is characterized by design homogeneity and short-term outcome indicators. Priority directions include the following: (1) validating predictive evidence of WBA tools for independent practice competency; (2) assessing the effects of Milestones 2.0 equity improvement; (3) conducting qualitative analysis of narrative comments; and (4) validating the effectiveness of the consortium system in the Chinese context.

## 9 Conclusion

This review synthesized three dimensions of CBME assessment in anesthesiology: tool validity evidence, equity, and faculty readiness. WBA tool validity evidence remains in the early stages, with most studies being single-center, small-sample designs, and patient safety outcome indicators virtually unadopted (31).

Sun et al. (15) and Matava et al. (16) collectively revealed that assessment differences originate from both group-level structural inequality and individual-level rater heterogeneity. Bailey et al. (55) showed that 64% of pediatric anesthesia faculty had never received formal CBME training, with faculty readiness deficiency constituting a significant implementation bottleneck. The Chinese consortium system has initiated localized exploration, but predictive evidence still requires verification.

Future research should prioritize filling two gaps: longitudinal tracking of WBA predictive evidence and specialty-specific randomized controlled trials of equity intervention effects. Clinical educators should establish routine rater calibration mechanisms and incorporate feedback quality into performance evaluations. Policymakers should mandate CBME training in continuing education and set ceilings on administrative burden. The success of CBME assessment in anesthesiology relies on a dynamic equilibrium among validity, equity, and feasibility. This narrative review is limited by the absence of a systematic literature search and the integration of quantitative data. The relative weights of individual components may vary due to interindividual differences and institutional contexts, and their interactions may exhibit non-linear characteristics.
